# Author Correction: Robust cytoplasmic partitioning by solving a cytoskeletal instability

**DOI:** 10.1038/s41586-026-10390-1

**Published:** 2026-04-22

**Authors:** Melissa Rinaldin, Alison Kickuth, Adam Lamson, Benjamin Dalton, Yitong Xu, Pavel Mejstřík, Stefano Di Talia, Jan Brugués

**Affiliations:** 1 Cluster of Excellence Physics of Life, TU Dresden, Dresden, Germany; 2 Max Planck Institute of Molecular Cell Biology and Genetics, Dresden, Germany; 3 Fachbereich Physik, Freie Universität Berlin, Berlin, Germany; 4 Department of Cell Biology, Duke University Medical Center, Durham, NC USA; 5 Max Planck Institute for the Physics of Complex Systems, Dresden, Germany; 6 Present Address: Program in Developmental Biology, Sloan Kettering Institute, Memorial Sloan Kettering Cancer Center, New York, NY USA

**Keywords:** Microtubules, Embryogenesis, Biological physics, Embryology, Cell division

Correction to: *Nature* 10.1038/s41586-025-10023-z Published online 28 January 2026

In the originally published version of the article, there were two inadvertent plotting errors. In two graphs, while the original data are plotted correctly on the left curves (Fig. 1l and Extended Data Fig. 8b), the profiles were visually mirrored to help in the interface visualization, inadvertently introducing plotting errors.

In Fig. 1l, the confidence interval of the mirrored simulated data, represented by a grey shaded area, was erroneously shifted along the *x*-axis. In Extended Data Fig. 8b, a point was missing from the plot in the mirrored simulated profile. These errors were purely aesthetic and did not affect the data, results or conclusions of the study. The figures and their associated source data have been updated in the HTML and PDF versions of this article. For comparison, the original and corrected figures are available as Supplementary Information accompanying this amendment.

**Supplementary information** is available in the online version of this amendment.

## Supplementary information

Original and revised Fig. 1l and Extended Data Fig. 8b

