# Supplementary material for: Robust cytoplasmic partitioning by solving a cytoskeletal instability
Source: Nature. 2026 Jan 28;651(8105):501–10. doi: 10.1038/s41586-025-10023-z (PMC12979205; doi:10.1038/s41586-025-10023-z)
Supplement: Supplementary file 1 — Supplementary Notes 1–3 and Supplementary Tables 1–7 [file 41586_2025_10023_MOESM1_ESM.pdf]

nature portfolio

https://doi.org/10.1038/s41586-025-10023-z

Supplementary information

Robust cytoplasmic partitioning by solving a cytoskeletal instability

In the format provided by the authors and unedited

Supplementary Information
Robust cytoplasmic partitioning by solving a cytoskeletal instability

Melissa Rinaldin[1,2,*], Alison Kickuth[1,2,⊥], Adam Lamson[1,⊥],
Benjamin Dalton[4], Yitong Xu[5], Pavel Mejstřík[2],
Stefano Di Talia[5], and Jan Brugués[1,2,3,*]

[1]Cluster of Excellence Physics of Life, TU Dresden, Dresden, 01307 Germany
[2]Max Planck Institute of Molecular Cell Biology and Genetics, Dresden, 01307 Germany
[3]Max Planck Institute for the Physics of Complex Systems
[4]Fachbereich Physik, Freie Universität Berlin, 14195 Berlin, Germany
[5]Department of Cell Biology, Duke University Medical Center, Durham, NC 27710 USA
[6]Current address: Program in Developmental Biology, Sloan Kettering Institute,
Memorial Sloan Kettering Cancer Center, New York, NY, 10065, USA.

⊥ Equal contribution
∗ Corresponding author: melissa.rinaldin@tu-dresden.de, jan.brugues@tu-dresden.de

1 Supplementary Note 1: Quantification and statistical analysis

1.1 EB1 comets analysis

Time sequences of microtubule plus ends were first processed by using the registration plugin in Fiji *Stackreg* to remove global translations and rotations of the comets. Then a rectangular region of interest (ROI) corresponding to the aster-aster interaction zone was cropped. The aster-aster interaction region extended from close to the centrosomes (or bead) of one aster to close to the centrosomes (or bead) of the adjacent aster. The ROI was analyzed using a custom-made *Jython* script run in Fiji using tools of *Trackmate* to track the positions of the plus ends (**Extended Data Fig. 2a**). The resulting tracks were then analysed with a custom made script in Python to calculate their speed and direction. Following an image analysis method reported previously [16], the ROI was then divided in a grid of bins. In each bin in the grid, the amount of tracks going to left and to the right were quantified. Tracks going from left to right were considered to belong to the left aster and vice versa. Then, the bins were averaged in the y direction (**Extended Data Fig. 2b**) and the amount of tracks belonging to the two asters was plotted in function of the distance between centrosomes (or beads) of the asters. Since the orientation of the aster is random, one of the aster profile was inverted so that for each experiments two profiles overlapped. Multiple profiles were then averaged (**Extended Data Fig. 2d**). We note that the shape of the density profile from the EB1 comets follows the one of the fluorescence intensity of the tubulin, with the advantages that (1) the EB1 comet analysis allows to distinguish the microtubules of the two asters and (2) provides the absolute number of microtubules. To verify the initial decay that is expected in 2D close to the centrosomes (**Extended Data Fig. 2e**), we analyzed the radial density of comets of one aster (**Extended Data Fig. 2g**). The image was divided in circular arcs with the aster center as the circle center and the density of microtubules averaged across the arcs. In this region, the directionality of the comets was ignored since all the comets belong to the analysed aster (**Extended Data Fig. 2f**).

1.2 Invasion time analysis

Time sequences of microtubules for arrested extract were acquired. Videos that were taken with the 4x objective are representative of the entire aster structure in z. If the magnification was higher than 4x, the videos were acquired as z-stack since the asters occupied about 200 μm in height and their structure had different features along z. These videos were converted into maximum-intensity projections in Fiji. All these videos were pre-processed as follows:

(1) The invasion event was selected by cropping the Supplementary Video in space and time (**Extended Data Fig. 7a**).

(2) The global translations and rotations were eliminated with Fiji plugin *Stackreg*.

(3) The background was subtracted. Depending on the resolution, a window ranging from 25 to 50 pixel size was used.

(4) A Gaussian blur of 2 pixel size was applied to remove noise.
Then, the microtubule intensity over the line connecting the centers of the asters was acquired for the entire invasion event with a custom-made Fiji script using *PlotProfile* (yellow line in **Extended Data Fig. 7a** and profiles in **Extended Data Fig. 7b**). These values were then analyzed with a custom-made script in Python performing the following:
(1) The intensity values were fitted with the following function:

$$f(x) = A_1 \exp - \frac{(x-b_1)^2}{2c_1^2} + A_2 \exp - \frac{(x-b_2)^2}{2c_2^2} + (dx+e),$$

where the two Gaussian functions are used to fit the density profiles of the two asters from the centrosomes to the aster-aster interaction zone and linear function corrects for the fact that the intensity at the centrosomes of the asters is not systematically equal among the asters, probably because of non-uniform illumination (**Extended Data Fig. 7c**).
(2) The background was removed by subtracting to the fits the minimum value of the fitted curves.
(3) The boundary between the asters was found by calculating the point where the two Gaussians crossed. For each Gaussian function defined from the centrosomes to the point of crossing, the underlying area was measured numerically using Simpson's rule. This area corresponds to the aster mass.
(4) The invasion time t_{inv} was defined as the time when:

$$M_{invaded}(t_{inv}) = \frac{M_{invaded}(t_0)}{e},$$

where $M_{invaded}(t)$ is the mass of the invaded aster over time and t_0 corresponds to the time that the asters start interacting **Extended Data Fig. 7d** .
(5) The plot in **Fig. 2d** in the main text was then obtained by plotting the invasion time versus the normalized difference of mass of the two asters at a time equal to t_0:

$$norm(\Delta M) = \frac{|M_1 - M_2|}{M_1 + M_2}.$$

1.3 Quantification of microtubule dynamics

Polymerization speed For all organisms, polymerization speed v_p was measured by averaging the values of the speed of single plus ends tracks found with a custom-made *Jython* script using tools of *Trackmate*.
Microtubule turnover Microtubule turnover was measured by following tubulin speckles in cycling extract and by performing fluorescence recovery after photobleaching (FRAP) and photo-conversion experiments in embryos and in extract with added centrosomes. While ideally speckle experiments are the most appropriate choice for this type of measurement, they were not possible to perform in embryos and for extract with added centrosomes because of the high

noise of tubulin, sparse structure microtubules in asters, and longer times required for aster formation (extract with centrosome case).

Speckle analysis Analysis of the tracks of single tubulin monomers was performed with with a custom-made *Jython* script base using tools of *Trackmate*. Lifetimes of tubulin were measured, and their relative frequency was fitted with the following function:

$$f(t) = At^{-3/2}\exp(-t/\tau),$$

where τ is four times the expected lifetime of a microtubule of average length. The turnover θ in the unit of min^{-1} is defined as

$$\theta = \frac{60}{\tau/4}.$$

FRAP analysis Microtubule turnover was measured by analyzing the FRAP (zebrafish and centrosomes in extract) and photo-conversion (*Drosophila*). The recovery signal was analyzed with a custom-made Python script. Briefly, for each ROI, the data were normalized such that the intensity just after the bleaching was zero, as follows:

$$I_{corr}(t) = \frac{I(t) - I_{tbleach}}{I(t_0) - I_{tbleach}}.$$

$I_{corr}(t)$ is the corrected intensity over time, $I(t)$ is the intensity over time, $I(t_0)$ is the intensity at the time t_0, and $I_{tbleach}$ is the intensity just after the bleaching. $I_{corr}(t)$ curves were fitted with the following function:

$$I_{corr}(t) = A(1 - \exp(-tk)) + B,$$

where k is an estimation of the microtubule turnover.

1.4 Compartment segmentation, cell cycle time, and cytoplasmic volume fraction quantification

Time lapses of cytoplasmic extract were acquired. The fluorescence signal of the lipids showing the cytoplasmic compartments was used for the segmentation. Images were pre-processed to remove background and then analyzed using a custom-made Python Script using the segmentation algorithm *Cellpose* [55]. Cell cycle duration was quantified by measuring the period of oscillations of compartment size and double-checked by visually inspecting the videos. Cytoplasmic volume fractions in Figure 5e were found by first measuring the axis length of the cytoplasmic region covered by the aster in the 2D images with Fiji using ROI tool measurements. The volume was then found by multiplying the values obtained with the known thickness of the same (for extract) or third axis for embryos. The volumes were normalized by total volume of the field of view.

2 Supplementary Note 2: Theory of aster-aster interactions

Here we consider a theoretical description of the growth of two interacting asters. For simplicity, we consider a 1D description of the problem where the densities of the two asters are ρ_1 and ρ_2, and consider only the populations of microtubules facing the two asters (**Fig. 1k**). We also consider that both asters have access to a common pool of nucleators that is quickly equilibrated compared to the growth dynamics of the asters. The density of microtubules propagates as an autocatalytic wave away from the center of the aster [15], which is inhibited by the interaction of microtubules of opposed polarity from the other aster:

$$\frac{d}{dt}\rho_i = \mp v_p \partial_x \rho_i + k_{bra} n_{bi} - \theta \rho_i - \lambda \frac{\rho_1 \rho_2}{\rho_1 + \rho_2}, \tag{1}$$

$$\frac{d}{dt} n_{bi} = k_b l_b n_u \rho_i - k_u n_{bi}, \tag{2}$$

where ρ_i ($i = 1, 2$) refers to the two microtubule densities, n_{bi} ($i = 1, 2$) refers to the bound nucleators to each microtubule population, and k_{bra} the corresponding nucleation rate. v_p is the velocity of microtubule polymerization, θ is the microtubule turnover rate, and λ is the strength of inhibition at antiparallel overlaps. n_u is the density of unbound nucleators, k_b is a binding rate, l_b is a characteristic binding length scale for the active nucleators, and k_u is the unbinding rate from microtubules for bound nucleators. We note that any arbitrary function $f(\rho_1, \rho_2)$ acting as inhibition, that is symmetric with respect to both densities (i.e., inhibiting the growth or increasing turnover of both networks equally), would lead to the same stability criterium of the boundary (see section below). We assume the binding and unbinding kinetics of nucleators are fast compared to the growth dynamics of the asters [15],[56, 57], and that the total amount of nucleators is conserved ($n_u + n_b = n_t$), implying:

$$n_{bi} = \frac{k_b l_b}{k_u} n_u \rho_i = \frac{k_b l_b}{k_u} (n_t - n_{b1} - n_{b2}) \rho_i. \tag{3}$$

Solving these two equations leads to the bound population of nucleators for each microtubule population:

$$n_{bi} = \frac{k_b l_b}{k_u} \frac{n_t \rho_i}{1 + \frac{k_b l_b}{k_u} (\rho_1 + \rho_2)}. \tag{4}$$

Substituting the population of bound nucleators into equation (1), leads to the dynamics of aster interaction:

$$\frac{d}{dt}\rho_i = \mp v_p \partial_x \rho_i + \alpha \frac{\rho_i}{1 + (\rho_1 + \rho_2)/\rho_s} - \theta \rho_i - \lambda \frac{\rho_1 \rho_2}{\rho_1 + \rho_2}, \tag{5}$$

where $\alpha = \frac{k_b l_b}{k_u} k_{bra} n_t$ characterizes the rate of autocatalytic growth at low density of microtubules, $\alpha\rho_i$, and $\rho_s = \frac{k_u}{k_b l_b}$ is a density of microtubules that indicates the saturation of microtubule nucleation, $\alpha\rho_s$, due to depletion of nucleators as they bind to microtubules. The resulting nucleation term is reminiscent of a Michaelis-Menten kinetics, where the microtubules and nucleators would correspond to the substrate and enzyme.

2.1 Linear stability analysis

Here we consider the stability of the solutions. Since the stability analysis will depend on the steady-state solutions, which we cannot obtain analytically, here we will analyse whether the boundary between both densities is stable. For that, we consider changes in the value of densities around ρ_{int}, which is the point at which both densities are equal at the overall region, and consider changes in the differences between the two networks, $\rho_1 - \rho_2 = \rho_1^0 - \rho_2^0 + \delta\rho$, where ρ_1^0 and ρ_2^0 are the solutions at steady state. Around ρ_{int}, $\rho_1 - \rho_2 = \delta\rho$, and we consider perturbations on the density that add up to 0 at this point, corresponding to moving the front, $\rho_1 = \rho_1^0 + 1/2\delta\rho$, and $\rho_2 = \rho_2^0 - 1/2\delta\rho$. For any arbitrary inhibition function $f(\rho_1, \rho_2)$, linear stability of the interface leads to a term $\frac{1}{2}\delta\rho(\frac{\partial f}{\partial \rho_1} - \frac{\partial f}{\partial \rho_2})$, which vanishes for symmetric functions with respect to ρ_1 and ρ_2 (i.e., inhibiting the growth or increasing turnover of both networks equally). This leads to an evolution of the perturbation at the interface at leading order with the perturbation $\delta\rho$ that reads:

$$\frac{d}{dt}\delta\rho = -v_p(\partial_x\rho_1 + \partial_x\rho_2) + \frac{\alpha\delta\rho}{1 + 2\rho_{int}/\rho_s} - \theta\delta\rho. \tag{6}$$

$$= \left(\frac{\alpha}{1 + 2\rho_{int}/\rho_s} - \theta\right)\delta\rho \equiv \epsilon\delta\rho, \tag{7}$$

where the first term cancels at the interface because of symmetry. Thus, the interface is stable if $\epsilon < 0$, leading to the stability criterium:

$$\theta > \frac{\alpha}{1 + 2\rho_{int}/\rho_s}. \tag{8}$$

2.2 Estimation of aster-aster interaction parameters

To estimate the mean and error of the parameters, α, λ, ρ_0, and ρ_s, of the 1D model, we performed a bootstrap analysis of all experimental spatial profiles (**Fig. 1l, 3c,e,g, 4e,k, Extended Data Fig. 8b,e,f,h**). We generate $M = 1000$ sample profiles using the mean and standard error of the binned data points. We perform a global fit of these generated profiles to the system of equations from equation (5) steady-state,

$$\partial_x\rho_i = \pm v_p^{-1}\left(\alpha\frac{\rho_i}{1 + (\rho_1 + \rho_2)/\rho_s} - \theta\rho_i - \lambda\frac{\rho_1\rho_2}{\rho_1 + \rho_2}\right). \tag{9}$$

Using the `scipy` computational suite, optimization was carried out using the `basinhopping` algorithm while the steady-state boundary value problem of equation (9) was solved using `solve_bpv`. The use of basin hopping [58] produced the best fits as it avoided getting stuck in local minima. We used the cost function

$$\mathcal{L} = \sum_{i=1}^{2}\sum_{n=1}^{N}(\rho_i(x_n) - \rho_i^{bt}(x_n))^2, \tag{10}$$

where N is the number of data points from the experimental profiles, x_n are the locations of the data points, and ρ_i^{bt} and ρ_i are the bootstrapped and fitted profiles, respectively.

We note that multiple steady-states exist in the unstable cases, though we sought the unstable equilibrium where the solutions are symmetric around the center between nucleating bodies. As such, we do not consider solutions to parameter sets where $|\rho_1(x_{int}) - \rho_2(x_{int})| > .05 \cdot \max(\rho_i)$. We further filter poorly fitting solutions by rejecting parameters sets with cost functions in the top 5% of fitted data.

2.3 Angular and higher dimensionality continuum model

We can extend our 1D mean-field model to higher dimensions by introducing an aster MT distribution function $\psi_i(\boldsymbol{x}, \hat{\boldsymbol{p}}, t)$ which accounts for the MT orientation/direction of growth $\hat{\boldsymbol{p}}$ and position vector $\boldsymbol{x} = (x, y, z)^T$. This maps back onto the 1D model via the constraint

$$\rho_i(x,t) = \oint d\hat{\boldsymbol{p}}\psi(\boldsymbol{x}, \hat{\boldsymbol{p}}, t) \tag{11}$$

and assuming that ψ_i does not vary in the $\hat{\boldsymbol{y}}$- and $\hat{\boldsymbol{z}}$-directions, i.e., the directions lying within the plane of the aster interface. The integral is a closed contour (or surface) integral over the unit circle (or sphere), depending on the system's dimensionality.

We must also introduce a new distribution function for bound nucleators $\eta_i(\boldsymbol{x}, \hat{\boldsymbol{p}}, t)$ that accounts for bound MT directionality, since the orientation of newly nucleated MTs depends on that of the MT to which a nucleator is bound. This comes with a normalization condition similar to Eq.11

$$n_{bi}(x,t) = \oint d\hat{\boldsymbol{p}}\, \eta_i(\boldsymbol{x}, \hat{\boldsymbol{p}}, t). \tag{12}$$

We write the analogous dynamical equations to Eqs.(1)

$$\frac{d}{dt}\psi_i = -\nabla \cdot (\boldsymbol{v}\psi_i) + k_{bra} \oint d\hat{\boldsymbol{p}}' \eta_{bi}(\hat{\boldsymbol{p}}')\mathcal{G}(\hat{\boldsymbol{p}}, \hat{\boldsymbol{p}}', \sigma_{bra}) - \Theta\psi_i - \lambda \frac{\oint d\hat{\boldsymbol{p}}'\psi_i(\hat{\boldsymbol{p}})\psi(\hat{\boldsymbol{p}}')f(\hat{\boldsymbol{p}} \cdot \hat{\boldsymbol{p}}')}{\oint d\hat{\boldsymbol{p}}'\psi(\hat{\boldsymbol{p}}')} \tag{13}$$

$$\frac{d}{dt}\eta_i = k_b l_b n_u \psi_i - k_u \eta_i \tag{14}$$

$$\frac{d}{dt}n_u = \sum_i \oint d\hat{\boldsymbol{p}}\left[k_u\eta_i(\hat{\boldsymbol{p}}) - k_b l_b n_u \psi_i(\hat{\boldsymbol{p}})\right], \tag{15}$$

where $\boldsymbol{v} = v_p\hat{\boldsymbol{p}}$ is the MT velocity and $\psi = \sum_i \psi_i$ is the total MT distribution function. The function $f(\hat{\boldsymbol{p}} \cdot \hat{\boldsymbol{p}}')$ introduces an angular dependence to inhibition. The second term on the RHS accounts for was the branching angular distribution observed in experiments[59] via the nucleating kernel

$$\mathcal{G}(\hat{\boldsymbol{p}}, \hat{\boldsymbol{p}}', \sigma_{bra}) = A \exp\left(\frac{-\cos^{-1}(\hat{\boldsymbol{p}} \cdot \hat{\boldsymbol{p}}')^2}{2\sigma_{bra}^2}\right), \tag{16}$$

where $A = \oint d\hat{\boldsymbol{p}} \exp\left(\frac{-\cos^{-1}(\hat{\boldsymbol{p}} \cdot \hat{\boldsymbol{z}}')^2}{2\sigma_{bra}^2}\right)$ is a normalizing constant dependent on the dimensionality such that $1 = \oint d\hat{\boldsymbol{p}}\mathcal{G}(\hat{\boldsymbol{p}}, \hat{\boldsymbol{p}}', \sigma_{bra})$. We do not include angular flux into the equation – aside from nucleation – as we assume that angular diffusion or advection is negligible compared to the other timescales.

We now examine how asters of higher dimensionality inhibit each other at the interface. We do this by assuming a symmetric distribution of microtubules near the interface of the two and marginalizing Eq.(13) over $\hat{\boldsymbol{p}}$. This gives an equation for ρ_i that has terms equatable to the 1D model and therefore can compare the different inhibitory, growth and nucleation parameters. The resulting equation is:

$$\frac{d}{dt}\rho_i = -v_g \oint d\hat{\boldsymbol{p}}(\hat{\boldsymbol{p}}\cdot\hat{\boldsymbol{x}}\partial_x\psi_i) + k_{bra} \oint d\hat{\boldsymbol{p}} \oint d\hat{\boldsymbol{p}}'\eta_i(\hat{\boldsymbol{p}}')\mathcal{G}(\hat{\boldsymbol{p}}, \hat{\boldsymbol{p}}', \sigma_{bra}) - \Theta\rho_i - \lambda\frac{\oint d\hat{\boldsymbol{p}} \oint d\hat{\boldsymbol{p}}'\psi_i(\hat{\boldsymbol{p}})\psi(\hat{\boldsymbol{p}}')f(\hat{\boldsymbol{p}} \cdot \hat{\boldsymbol{p}}')}{\sum_j \rho_j}, \tag{17}$$

where we have used Eq.(11).

Next, from Extended Data Fig. 3e, we notice that the angular distribution at the boundary of simulated asters remains remarkably constant over time. We therefore make the ansatz for the distribution of ψ: $\psi_i(x, \hat{\boldsymbol{p}}, t) = \rho_i(x, t)\xi_i(\hat{\boldsymbol{p}})$, where we show that ξ_i takes a profile like that of the branching kernel with a different standard deviation $\sigma_\xi = 0.260\,\mathrm{rad}$ and $\hat{\boldsymbol{p}}' = \hat{\boldsymbol{x}}$, i.e., $\xi(\hat{\boldsymbol{p}}) = \mathcal{G}(\hat{\boldsymbol{p}}, \hat{\boldsymbol{x}}_i, \sigma_\xi)$ (See figure Extended Data Fig. 3e-f). The unit vector $\hat{\boldsymbol{x}}_i$ points away from centrosome i .

In the regime where nucleator binding kinetics are fast compared to the dynamics of ψ_i, we see from Eq.(14) that η_i shares the same angular distribution: $\eta_i(x, \hat{\boldsymbol{p}}, t) = \frac{k_u}{k_b l_b n_u}\rho_i\xi_i$. Continuing to assume a quasi-steady state for bound nucleators, with Eq.(15) and $\rho_i = \oint d\hat{\boldsymbol{p}}\psi_i$, we can further reduce the system by solving for n_u:

$$n_u = n_t - \sum_i \oint d\hat{\boldsymbol{p}}\,\eta_i = n_t - \frac{k_b l_b}{k_u} n_u \sum_i \oint d\hat{\boldsymbol{p}}\,\psi_i \tag{18}$$

$$n_u = \frac{n_t}{1 + \sum_i \rho_i/\rho_s}, \tag{19}$$

where we have used the definition for ρ_s from Eq.5. This then leads to

$$\eta_i = \frac{n_t}{\rho_s + \sum_j \rho_j}\xi_i\rho_i. \tag{20}$$

Substituting Eq.(20) and Eq.(19) into Eq.(13)

$$\frac{d}{dt}\rho_i = -v_g\left(\oint d\hat{\boldsymbol{p}}\,\hat{\boldsymbol{p}}\cdot\hat{\boldsymbol{x}}\xi_i\right)\partial_x\rho_i + \frac{k_{bra}n_t}{\rho_s+\sum_j\rho_j}\left(\oint d\hat{\boldsymbol{p}}\oint d\hat{\boldsymbol{p}}'\xi_i(\hat{\boldsymbol{p}}')\mathcal{G}(\hat{\boldsymbol{p}},\hat{\boldsymbol{p}}',\sigma_{bra})\right)\rho_i - \Theta\rho_i - \lambda\rho_i\frac{\sum_j\left(\oint d\hat{\boldsymbol{p}}\oint d\hat{\boldsymbol{p}}'\xi_i(\hat{\boldsymbol{p}})\xi_j(\hat{\boldsymbol{p}}')f(\hat{\boldsymbol{p}}\cdot\hat{\boldsymbol{p}}')\right)\rho_j}{\sum_j\rho_j}.$$

Examining the above equations, we notice several constants emerge that renormalize the velocity, branching, and inhibition terms with respect to the 1D model:

$$\frac{d}{dt}\rho_i = -v_g a\partial_x\rho_i + \frac{k_{bra}n_t}{\rho_s+\sum_j\rho_j}\rho_i - \Theta\rho_i - \lambda\rho_i\frac{\sum_j c_{ij}\rho_j}{\sum_j\rho_j}. \tag{21}$$

where the second the integral is identically 1 because of the normalization of the functions $\mathcal{G}$ and ξ_i. The constants a and c_{ij} are

$$a = \left(\oint d\hat{\boldsymbol{p}}\,\hat{\boldsymbol{p}}\cdot\hat{\boldsymbol{x}}\,\xi_i\right) \tag{22}$$

$$c_{ij}(\hat{\boldsymbol{x}}_i,\hat{\boldsymbol{x}}_j) = \oint d\hat{\boldsymbol{p}}\oint d\hat{\boldsymbol{p}}'\xi_i(\hat{\boldsymbol{p}})\xi_j(\hat{\boldsymbol{p}}')f(\hat{\boldsymbol{p}}\cdot\hat{\boldsymbol{p}}'). \tag{23}$$

In the case of only two asters, we can compare directly to Eq.(5)

$$\frac{d}{dt}\rho_i = \mp\tilde{v}_g\partial_x\rho_i + \frac{\alpha}{1+(\rho_1+\rho_2)/\rho_s}\rho_i - \Theta\rho_i - \tilde{\lambda}\frac{\rho_1\rho_2}{\rho_1+\rho_2} - \gamma\frac{\rho_i^2}{\rho_1+\rho_2}, \tag{24}$$

where $\tilde{v}_g = av_g$ and $\tilde{\lambda} = c_{12}\lambda$ are the renormalized parameters. In this equation, we now see that an extra quadratic term in ρ_i has emerged, accounting for possible self-inhibition of MTs from the same aster, with a prefactor $\gamma = c_{ii}\lambda$. This allows to control self vs aster-aster inhibition. Because of the values of σ_ξ and simulation values for the inhibitory steepness parameter β, this term is orders of magnitude smaller than other inhibitory effects or turnover, as expected (**Extended Data Fig. 3f**).

Far from the inhibition zone, and close to the center of the compartment, the dimensionality affects how the microtubule density decays away from the center through the flux term, which becomes $\nabla(v_p\hat{r}\rho) = ((d-1)v_p\rho/r + v_p\partial_r\rho)\,\hat{r}$, where we are using the radial symmetry of an aster, and d is the dimension we are considering. This implies that density decreases like $1/r$ in 2 and 3 dimensions simply because of the radial symmetry of the aster and the radial velocity of polimerization. This term adds to the turnover term $-\Theta\rho$ and together act as a modified turnover rate. We note that the geometrical effect becomes smaller than turnover for a radius $r > (d-1)v_p/\Theta \sim (d-1)5\mu m$. As shown in **Extended Data Fig. 2e**, the effect of geometry when fitting the densities is restricted to very close to the centrosome. As a consequence, we use the 1D model for fitting all conditions and studying the stability.

3 Supplementary Note 3: Agent-based modeling

3.1 2D simulations with nucleator diffusion for planar channel geometry and invasion time analysis

We combine 2D microtubule filaments with a source of diffusing nucleators, which mediate autocatalytic microtubule nucleation, and inhibitory interactions. Microtubule filaments are represented as lines on a 2D plane with a center of mass vector $\mathbf{r}_i$ for the ith filament and an orientation unit vector $\hat{\boldsymbol{p}}_i$. Microtubule minus-ends and orientations are static. The length of each filament L_i changes at a rate of $v_p = 21$ µmmin^{-1} for polymerization and $v_d =$ -33 µmmin^{-1} for depolymerization [15]. Catastrophe events occurs at a rate of $k_c = 2.5$ min^{-1} [15], and we ignore rescue events. We simulate a finite pool of N_n nucleators, such that unbound nucleators exhibit Brownian diffusion with displacement variance $\sigma = \sqrt{2\Delta t D_{nuc}}$, where $D_{nuc} =$ 1 µm^{2}s^{-1} is the diffusion coefficient and Δt =100 μs is the simulation step size. Unbound nucleators can bind to a neighboring filament at a rate $k_b = 3{\times}10^{-3}$ min$^{-1}\mu$m^{-1}, such that the probability of binding depends on the total length of filaments in the systems, and unbind at a constant rate of $k_u = 3$ min^{-1} per nucleator. N_n, k_b, and k_u are selected to optimize the agreement between simulation profiles and experimental results. When bound to a mother filament, nucleators can generate a new daughter filament at a rate of $k_d = 0.82$ s^{-1}. Daughter filaments are nucleated with a normally distributed orientation form the mother filament, with a mean angle of 0° and a standard deviation of 9° [59]. Upon generating a new filament, nucleators remain bound until either the daughter filament fully depolymerizes, or until the mother filament has depolymerizes past the attachment point. Nucleators are returned to an initially-inactive unbound state and are reactivated with a rate $k_a = 2.5$ s^{-1}, chosen to ensure that nucleators have enough time to diffuse away from their previous binding site before immediately rebinding (**Extended Data Fig. 3a** and **Supplementary Video 15**). Simulations were performed using a custom-made C++ code. All analysis was performed using custom-written MATLAB scripts.

Inhibition: When the growing tip of a polymerizing filament meets another filament, the probability that the approaching filament will be switched to a depolymerizing state, or *inhibited*, depends on the angle of incidence θ (**Extended data Fig. 3c-d**). The rate at which that the approaching filament will undergo an inhibition event is specified by

$$k_\lambda(\theta) = \frac{\lambda_{2D}}{1 + e^{-\beta(\theta/\pi - 0.5)}}, \tag{25}$$

where β is the steepness of the sigmoidal function, which can be tuned to increase the range of inhibition and which we set to be $\beta = 22$. The 2D inhibition prefactor λ_{2d} is set to $8 \times 10^5\,\text{min}^{-1}$ so that there is a 50% chance a filament depolymerizes when encountering perpendicular filament $P(\theta) = 1 - \exp(-k_\lambda \Delta t)$. Here, $\theta = \arccos(\hat{\boldsymbol{p}}_a \cdot \hat{\boldsymbol{p}}_b)$, where $\hat{\boldsymbol{p}}_a$ is the orientation vector of the approaching filament and $\hat{\boldsymbol{p}}_b$ is the orientation vector of the second

filament.

Planar-channel simulations: The 2D Planar channels are 200 μm long and 50 μm wide (**Extended Data Fig. 7f**). Unbound nucleators diffuse freely with periodic boundary conditions. We include bulk microtubule nucleation at the boundaries in the x-dimension. To simulate the unstable systems, we nucleate new microtubules with minus ends located at either $x = 0$ μm or $x = 200$ μm, at a rate of $k_n = 0.5$ s^{-1} such that orientation vectors are $\hat{\boldsymbol{p}}_i = [1, 0]$ and $\hat{\boldsymbol{p}}_i = [-1, 0]$, respectively. We simulate a total of $N_n = 800$ nucleators, such that the total nucleator density is 0.08 nucleators per μm^2. For the stable systems, we simulate in the same 2D channel, but we nucleate new microtubules with minus ends located at either $x = 75.25$ μm or $x = 124.75$ μm at a rate of $k_0 = 2.6$ s^{-1}. Again, the orientation vectors are $\hat{\boldsymbol{p}}_i = [1, 0]$ and $\hat{\boldsymbol{p}}_i = [-1, 0]$, respectively. We simulate a total of $N_n = 225$ nucleators, such that the total nucleator density is 0.0225 nucleators per μm^2. For comparison with EB1 experiments, microtubule densities refer to the density of growing microtubule plus-ends. For each system, we run a total of 40 individual simulations and average the results. For the unstable system, we begin collecting data once an interface between the two aster networks is well-formed, which occurs after approximately 25 minutes (**Extended Data Fig. 7g**). We filter data by removing the system for which the total EB1 mass differs significantly at $t = 25$ min (**Extended Data Fig. 7h**) For accepted systems, we collect data for 5 minutes at a rate of one frame every 5 seconds and accumulate EB1 histograms with bin spacings of 8 μm. The average density profile is shown in **Extended Data Fig. 7m**. The individual histograms for 21 accepted systems at frame $t = 25$ min are shown, with standard errors. The curve fitting, as presented in the main text, is shown in **Extended Data Fig. 7n**.

Invasion time simulations: Simulation results for the invasion time curve (**Fig. 2d**) are generated using the same 2D planar-channel geometry as described above. To simulate many invasion events, we begin simulations with two initial aster structures. Each structure begins with 150 microtubules such that initial lengths are exponentially distributed with a mean length of 7 μm. Initial microtubules are initiated in a polymerizing state and exhibit the usual turnover rules. We then generate an initial-aster mass difference ΔM_{init} by uniformly scaling the initial lengths of all filaments in one aster by ΔM_{init}. We run 30 planar-channel simulations for each $\Delta M_{init} = 1, 2, 3, 4,$ and 5. We select an invasion region as shown in (**Extended Data Fig. 7f**). The normalized mass difference $norm(\Delta M)$ (see section 1.2) is determined at the beginning of the invasion t_0 and an invasion time t_{inv} is defined as $M_{invaded}(t_{inv}) = M_{invaded}(t_0)/\text{e}$, as introduced in section 1.2.

3.2 Simulations with implicit nucleator reservoir for 3D aster geometry

To complement the experimental results presented in the main text, we developed a GPU-accelerated kinetic Monte Carlo (KMC) simulation framework to efficiently model microtubule

and nucleator dynamics in three dimensions. This approach allows us to incorporate key biological processes—such as microtubule growth, catastrophe, inhibition, and nucleation—while maintaining computational tractability at larger scales. The assumptions, algorithmic details, and stochastic event handling are described below, along with pseudocode outlining the main simulation steps (Algorithm 1). Parameters used throughout are summarized in Table 7.

This new simulation framework allows for greater numbers of microtubules (MT) and larger time steps compared to the method used for two-dimensional systems with explicitly modeled unbound nucleators (Section 3.1). This is achieved by assuming that unbound nucleators diffuse rapidly relative to other biological processes in the model, effectively maintaining a spatially homogeneous reservoir of nucleators.

Leveraging this assumption, we could rapidly explore parameter spaces and compare one-, two-, and three-dimensional models incorporating MT growth, turnover, inhibition, and nucleation, as well as nucleator binding kinetics. These simulations were used to produce **Fig. 1n,o** and **Fig. 2c** of the main text, demonstrating the robustness of aster instabilities across biophysically relevant dimensions.

Kinetic Monte Carlo scheme for microtubule and nucleator dynamics: A system's state is defined by a set of MTs M and nucleators N. Each MT m is characterized by its length l_m, minus-end position $\boldsymbol{x}_m$, orientation $\hat{\boldsymbol{p}}_m$, and polymerization state s_m (growing, shrinking, or paused). Nucleators n are described by a binding state b_n and, if bound, a position x_n along an MT.

At each simulation step k, the KMC algorithm updates the system's state. In the limit of fast nucleator diffusion, three stochastic processes are explicitly captured:

1. Catastrophe events induced by MT interactions (i.e., inhibition and spontaneous catastrophe),
2. Nucleator binding to MTs and nucleation of new MTs,
3. Nucleation of MTs from centrosomes.

Each process is governed by a rate that may depend on the current system state (see Algorithms 3, 2, and 5).

Deterministic dynamics, such as MT polymerization and depolymerization, are integrated numerically for computational efficiency. The evolution of MT length is given by:

$$l_m^{k+1} = l_m^k + v_{p(d)}\Delta t,$$

where v_p and v_d are the polymerization and depolymerization velocities, respectively, and Δt is the fixed simulation time step. MTs that polymerize beyond the simulation domain boundaries are transitioned into a paused state but continue to undergo stochastic catastrophes and inhibition according to standard rules.

Calculation of Catastrophe Probability (Algorithm 2): We compute the probability that a MT undergoes catastrophe during a simulation time step by first evaluating its tip position $\mathbf{x}_{\text{tip}}$. A base catastrophe rate k_c - the rate at which a single MT will undergo catastrophe in the absence of other MTs - is assigned to all MTs. If the MT's length l_m exceeds a critical threshold[1] $2r_\lambda$, additional inhibitory contributions are added to the cumulative catastrophe rate.

For each other MT m', the shortest distance $r_{mm'}$ between the tip of m and the body of m' is computed. If $r_{mm'}$ is less than an interaction radius r_λ, we increase the catastrophe rate dependent on the relative orientation of the two MTs. The inhibition strength follows a sigmoid function of the angle between MT directions, controlled by parameters λ and β(see Section3.1). After summing all contributions, the total catastrophe probability P_{cat} over the time step Δt is computed using single-event Poisson statistics as $P_{\text{cat}} = 1 - \exp(-k_{\text{tot}}\Delta t)$, where k_{tot} is the accumulated rate.

Nucleator Binding to Microtubules (Algorithm 3): Physically, stochastic binding of nucleators to MTs depends on the total number of binding sites and unbound nucleators in solution following the reaction

$$[\text{N}_u] + [\text{S}] \longrightarrow [\text{N}_b] + [\text{S}]$$

where $[\text{N}_u]$ is the unbound nucleator concentration, $[\text{S}]$ is the concentration of MT nucleator bindings sites, and $[\text{N}_b]$ is the concentration of bound nucleators. We assume that the binding reaction is irreversible and nucleators only unbind if the site they are bound to disappears through MT depolymerization. Notice, we have also decided to allow for multiple nucleators to bind to the same binding site. While perhaps not totally physical, this assumption greatly simplifies the calculation of binding statistics and is valid in the limiting case where $[\text{N}_b] + [\text{N}_u] \ll [\text{S}]$.

The total number of binding sites are proportional to the total MT surface area and thus proportional to the total MT length l_{tot}. With this, we calculate probability P_b that an unbound nucleator binds to any MT during a time step Δt using single event Poisson statistics $P_b = 1 - \exp(-k_b l_{\text{tot}} \Delta t)$, where k_b is the binding rate constant per unit length. Since nucleators can bind to the same sites, binding probabilities are independent and the number of nucleators that successfully bind, ΔN_b, follows a binomial distribution with N_u trials and success probability P_b. Each binding nucleator is then assigned to a specific MT m_b selected randomly with probability proportional to the MTs length l_m. The position along the chosen MT is uniformly sampled between the minus-end and plus-end locations. The last two steps follow because all binding sites are equally likely to be bound on a single MT.

Microtubule Nucleation from Bound Nucleators (Algorithm 4): The implicit nucleator model simplifies nucleator kinetics by reducing the different states of nucleators from three stages to two. However, this means in order to compare the results of the two models, we must find a mapping between the different parameters. The kinetics of the 2D model with rapid

[1]This critical threshold was implemented to prevent an overabundence of self-aster inhibition.

diffusion can be written as

$$\frac{dN_a}{dt} = k_a N_b \tag{26}$$

$$\frac{dN_b}{dt} = -k_a N_b - k_u N_b + k_b l_{tot} N_u \tag{27}$$

$$\frac{dN_u}{dt} = k_u N_b - k_b l_{tot} N_u. \tag{28}$$

where we have ignored MT depolymerization and therefore the unbinding of active nucleators N_a. Bound but inactive nucleators were introduced to account for the microtubule-dependent diffusion of nucleators. However, in the limit of rapid diffusion, we can ignore this state and have an effective combined binding-nucleating rate. The dynamic equations would then simplify to

$$\frac{dN_a}{dt} = k_{eff} l_{tot} N_u \tag{29}$$

$$\frac{dN_u}{dt} = -k_{eff} l_{tot} N_u. \tag{30}$$

Assuming the linearity $N_b \propto N_u$, we find the effective nucleating rate $k_{eff} = k_a k_b/(k_a + k_u)$. In our 3D simulations, we set k_{eff} directly.

As implied by the above system of ODEs, new MTs immediately nucleate from the set of nucleators that have just attached to existing MTs, denoted ΔN_b. For each bound nucleator n, a new MT is created with its minus-end anchored at the nucleator's position $\boldsymbol{x}_n$. The orientation of the newly nucleated MT, $\hat{\boldsymbol{p}}_n$, is sampled from a biased angular distribution centered around the direction $\hat{\boldsymbol{p}}_b$ of the MT to which the nucleator is bound. The probability of selecting a particular orientation is given by

$$P(\hat{\boldsymbol{p}}_n) \propto \exp\left(-\frac{\arccos(\hat{\boldsymbol{p}}_b \cdot \hat{\boldsymbol{p}}_n)^2}{2\sigma_{\mathrm{bra}}^2}\right),$$

where σ_{bra} controls the spread of the nucleation angle relative to the parent MT. The newly created MTs are initialized with a small fixed length $l_m = 2d$ and are added to the set of MTs for subsequent dynamic evolution.

Aster simulations Fig. 1q and 2c of the main text show time-lapse sequences of three-dimensional aster invasion events, generated using the agent-based simulation framework described here. These simulations replicate MTs within a rectangular slab of cytoplasm with dimensions $313\,\mathrm{\mu m} \times 313\,\mathrm{\mu m} \times 50\,\mathrm{\mu m}$. The slab's short axis was aligned with the $\hat{\boldsymbol{z}}$-direction to reflect the geometry present in experimental systems derived from Xenopus egg extracts.

Within this volume, two centrosome-like structures—each serving as an aster nucleation center—were positioned $221\,\mathrm{\mu m}$ apart along the slabs diagonal and centered on the z-axis. Each centrosome had a radius of $100\,\mathrm{nm}$ and was initially seeded with 17 MTs, each of length

Algorithm 1 Microtubule dynamic simulations with implicit nucleator reservoir

Load simulation parameters (see Table 7).
Initialize the locations and states of MTs M, nucleators N, and centrosomes C.
for $i = 1$ to total steps S **do**
for each MT m in M **do**
if m is growing **then**
$l_m \leftarrow l_m + v_p \Delta t$
else if m is shrinking **then**
$l_m \leftarrow l_m - v_d \Delta t$
end if
end for
Remove MTs with $l_m < d$ (MT diameter).
for each MT m in M **do**
if m is growing or paused **then**
Calculate catastrophe probability $P_{\text{cat}} \leftarrow \text{ComputeCatastropheProbability}(m, M, \text{params})$ (Algorithm 2).
Sample random number $r \sim U[0, 1]$.
if $r < P_{\text{cat}}$ **then**
Set m to shrinking.
end if
end if
if m is growing $\wedge$ m outside bounds **then**
Set m to paused.
end if
end for
$\Delta N_b \leftarrow \text{BindNucleators}(M, N, \text{params})$ (Algorithm 3).
Unbind nucleators if (i) bound MT depolymerizes past their attachment site, or (ii) the MT they are bound to is removed.
$M \leftarrow M + \text{NucleateMTsFromNucleators}(\Delta N_b, \text{params})$ (Algorithm 4).
$M \leftarrow M + \text{NucleateMTsFromCentrosomes}(C, \text{params})$ (Algorithm 5).
end for

Algorithm 2 ComputeCatastropheProbability

Require: MT m, set of MTs M, parameters `params`

Compute tip location: $\mathbf{x}_{\text{tip}} \leftarrow \mathbf{x}_m + \hat{\mathbf{p}}_m l_m$

Initialize total catastrophe rate: $k_{\text{tot}} \leftarrow k_c$

if $l_m > 2r_\lambda$ **then**

  for each m' in M **do**

    if $m' \neq m$ **then**

      Compute shortest distance $r_{mm'}$ between $\mathbf{x}_{\text{tip}}$ and MT m'

      if $r_{mm'} \leq r_\lambda$ **then**

        Update catastrophe rate:

$$k_{\text{tot}} \leftarrow k_{\text{tot}} + \frac{\lambda}{1 + \exp\left[-\beta\left(\frac{\arccos(\hat{\mathbf{p}}_m \cdot \hat{\mathbf{p}}_{m'})}{\pi} - 0.5\right)\right]}$$

      end if

    end if

  end for

end if

Compute catastrophe probability:

$$P_{\text{cat}} = 1 - \exp(-k_{\text{tot}} \Delta t)$$

return P_{cat}

Algorithm 3 BindNucleators

Require: Set of MTs M, nucleators N, parameters `params`

Identify unbound nucleators: $N_u \subseteq N$

Collect MT lengths: $\{l_m\}$

Compute total MT length: $l_{\text{tot}} \leftarrow \sum_m l_m$

Compute binding probability for a nucleator:

$$P_b = 1 - \exp(-k_b l_{\text{tot}} \Delta t)$$

Sample number of binding events:

$$|\Delta N_b| \sim \mathcal{B}i(|N_u|, P_b)$$

Select $\Delta N_b \subseteq N_u$

for each nucleator n in ΔN_b **do**

  Sample MT m_b to bind to (weighted by l_m)

  Sample binding position along m_b: $x_b \sim l_{m_b} \times U[0, 1]$

  Bind n to m_b at x_b

end for

return ΔN_b

Algorithm 4 NucleateMTsFromNucleators

Require: Newly bound nucleators ΔN_b, parameters `params`

Initialize new MT set: $\Delta M \leftarrow \{\}$

for each nucleator n in ΔN_b **do**

  Retrieve bound MT direction: $\hat{\mathbf{p}}_b$

  Sample a unit vector $\hat{\mathbf{p}}_n$ from distribution:

$$P(\hat{\mathbf{p}}_n) \propto \exp\left(-\frac{\arccos(\hat{\mathbf{p}}_b \cdot \hat{\mathbf{p}}_n)^2}{2\sigma_{\text{bra}}^2}\right)$$

  Create new MT m:

- Length: $l_m = 2d$
- Direction: $\hat{\mathbf{p}}_m = \hat{\mathbf{p}}_n$
- Minus-end position: $\mathbf{x}_m = \mathbf{x}_n$ (nucleator location)

  Add m to ΔM

end for

return ΔM

Algorithm 5 NucleateMTsFromCentrosomes

Require: Set of centrosomes C, parameters `params`

Initialize new MT set: $\Delta M \leftarrow \{\}$

for each centrosome c in C **do**

  Compute expected number of MTs:

$$\langle|\Delta M|\rangle = k_n \Delta t$$

  Sample number of MTs to nucleate:

$$|\Delta M_c| \sim \mathcal{P}o(\langle|\Delta M|\rangle)$$

  for $i = 1$ to $|\Delta M_c|$ **do**

    Sample a random unit vector $\hat{\mathbf{p}}_c$ (uniform on sphere)

    Retrieve position and radius of c: $\boldsymbol{x}_c$, R_c

    Create new MT m:

- Length: $l_m = 2d$
- Direction: $\hat{\mathbf{p}}_m = \hat{\mathbf{p}}_c$
- Minus-end position: $\mathbf{x}_m = \mathbf{x}_c + R_c \hat{\mathbf{p}}_c$

    Add m to ΔM

  end for

end for

return ΔM

$8.3\,\mu\mathrm{m}$. The minus ends of these MTs were uniformly distributed on the centrosome surface oriented radially outward from their respective centrosomes.

MT nucleation continued throughout the simulation at a constant rate of $k_0 = 0.5\,\mathrm{s}^{-1}$, occurring uniformly across the centrosome surface (as implemented in Algorithm 5). The total number of nucleators was set to $N_n = 10000$, chosen to reproduce experimentally observed MT density profiles. Simulation snapshots and movies were rendered using the `Paraview` software suite.

To analyze MT density, we examined all MT tips falling within a narrow, centered rectangular region of size $200\,\mu\mathrm{m} \times 10\,\mu\mathrm{m} \times 10\,\mu\mathrm{m}$. This region was oriented along the axis connecting the two asters, allowing us to measure density along the invasion path. For Figure 1q, which reports MT density and its confidence interval prior to invasion, we ran four independent simulations using different random number generator seeds and initial MT arrangements. To minimize the impact of short-timescale fluctuations, MT tip counts were averaged over a $2\,\mathrm{min}$ interval beginning at the $58\,\mathrm{min}$ time point.

55. Carsen Stringer et al. "Cellpose: a generalist algorithm for cellular segmentation". In: *Nat. Methods* 18.1 (2021), pp. 100–106.

56. Doogie Oh, Che-Hang Yu, and Daniel J. Needleman. "Spatial organization of the Ran pathway by microtubules in mitosis". In: *Proc. Natl. Acad. Sci. USA* 113.31 (2016), pp. 8729–8734.

57. Bryan Kaye et al. "Measuring and modeling polymer concentration profiles near spindle boundaries argues that spindle microtubules regulate their own nucleation". In: *New J. Phys.* 20.5 (2018), p. 055012.

58. Brian Olson et al. "Basin Hopping as a General and Versatile Optimization Framework for the Characterization of Biological Macromolecules". In: *Advances in Artificial Intelligence* 2012.1 (2012), p. 674832.

59. Akanksha Thawani et al. "Spatiotemporal organization of branched microtubule networks". In: *eLife* 8 (2019). Ed. by Raymond E Goldstein and Anna Akhmanova, e43890.

4 Supplementary Tables

Condition	Number of independent samples	Total number of frames
X. laevis extract with sperm	8	40
X. laevis extract with AurkA beads	7	60
X. laevis extract with AurkA beads and Ran	5	25
X. laevis extract with sperm and MCAK	5	25
X. laevis extract with HeLa centrosomes	6	30
X. laevis extract with *Drosophila* embryo centrosomes	9	45
zebrafish	4	20
Drosophila	8	1200

Supplementary Table 1: Number of independent samples (biological replicates) and frames analyzed for the different extract conditions and organisms (technical replicates).

Organism	Number of tracks for speed	Number of independent samples
X. laevis extract arrested	5864	7
X. laevis extract cycling	10115	4
X. laevis extract with AurkA beads	6655	5
X. laevis extract with AurkA beads and Ran	22225	30
X. laevis extract with sperm and MCAK	10039	15
X. laevis extract with HeLa centrosomes	18130	6
X. laevis extract with *Drosophila* embryo centrosomes	4817	3
zebrafish	9320	4
Drosophila	10053	4

Supplementary Table 2: Number of independent samples (biological replicates) and EB1 tracks analyzed (technical replicates).

Organism	Number of lifetimes or ROIs	Number of independent samples
X. laevis extract arrested	12640	6
X. laevis extract cycling	5473	3
X. laevis extract with AurkA beads	3149	4
X. laevis extract with AurkA beads and Ran	16218	7
X. laevis extract with sperm and MCAK	6724	4
X. laevis extract with HeLa centrosomes	3 (ROI)	3
X. laevis extract with *Drosophila* embryo centrosomes	4 (ROI)	4
zebrafish	7 (ROI)	7
Drosophila	4 (ROI)	4

Supplementary Table 3: Number of independent samples (biological replicates) and lifetimes or ROI (technical replicates for speckle and FRAP or photo-conversion).

**Experimentally measured parameters**		
**Case**	v_p [µm/min]	θ [min^{-1}]
**Frog sperm asters-arrested**	21.7 ± 4.4	4.1 ± 0.3
**Frog sperm asters-cycling**	23.3 ± 7.9	4.0 ± 0.5
**Frog AurkA bead asters**	20.7 ± 5.6	4.4 ± 0.6
**Frog RanQ69L AurkA bead asters**	19.0 ± 4.8	4.5 ± 0.4
***X. laevis* extract with sperm beads and MCAK**	17.3 ± 6.6	4.0 ± 0.1
***X. laevis* extract with HeLa centrosomes**	20.6 ± 4.8	4.2 ± 0.2
***X. laevis* extract with *Drosophila* centrosomes**	19.7 ± 4.8	4.7 ± 0.4
**zebrafish**	22.0 ± 7.2	4.4 ± 0.9
Drosophila	15.4 ± 7.0	5.2 ± 0.9

Supplementary Table 4: Measured speeds and turnovers.

Parameters from fits of the simulations			
**Case**	α [$\mu m^{-2} min^{-1}$]	ρ_s [μm^{-2}]	ρ_0 [μm^{-2}]
**Unstable Planar Channel**	5.27 ± 0.09	0.32 ± 0.04	0.0162 ± 0.0006
**Stable Planar Channel**	3.06 ± 0.04	no saturation	0.062 ± 0.001

Supplementary Table 5: Parameters from fits of the simulations

Parameters from theoretical fits					
**Case** (**Range**)	α [μm^{-2} min^{-1}] (0-100)	ρ_s [μm^{-2}] (.01-∞)	ρ_0 [μm^{-2}] (0-100)	λ [μm^{-2} min^{-1}] (0-100)	α' [μm^{-2} min^{-1}] (calculated)
**Zebrafish**	5.60 ± 1.04	0.29 ± 0.39	0.01 ± 0.06	3.43 ± 2.96	5.17 ± 0.96
**Frog RanQ68L AurkA bead asters**	5.27 ± 0.58	0.26 ± 0.44	0.00 ± 0.03	1.59 ± 0.63	4.95 ± 0.50
**Frog sperm asters**	5.49 ± 0.64	0.22 ± 0.08	0.01 ± 0.00	2.47 ± 0.61	4.08 ± 0.52
***Drosophila* centrosomes**	6.35 ± 1.84	0.04 ± 0.04	0.05 ± 0.01	0.60 ± 1.57	4.57 ± 0.37
**HeLa centrosomes**	4.42 ± 1.58	0.27 ± 0.85	0.09 ± 0.08	1.69 ± 1.71	3.96 ± 0.29
**Frog MCAK sperm asters**	4.51 ± 2.37	0.19 ± 0.53	0.08 ± 0.02	1.09 ± 0.61	3.72 ± 0.21
**Frog AurkA bead asters**	7.55 ± 5.15	0.03 ± 0.03	0.26 ± 0.24	1.81 ± 1.56	3.69 ± 0.81
Drosophila	8.35 ± 15.29	0.19 ± 0.46	0.99 ± 0.03	27.02 ± 12.41	3.10 ± 1.23

Supplementary Table 6: Ranges for the parameters of the theoretical model for the different datasets. The values are obtained from the bootstrap analysis. Fitting parameters were constrained to the ranges shown in the table header.

**Parameter**	**Symbol**	**Value**	**Note**
**Microtubule parameters**			
Microtubule diameter	d	$0.025\,\mu m$	
Polymerization velocity	v_p	$21\,\mu m\,min^{-1}$	
Depolymerization velocity	v_d	$-33\,\mu m\,min^{-1}$	
Catastrophe rate	k_c	$2.5\,min^{-1}$	
Inhibition radius	r_λ	$2\,\mu m$	Explored range: 0.025-$2\,\mu m$. See Extended Data Fig. 3g for parameter scan.
Inhibition strength	λ	$450\,min^{-1}$	Explored range: 38.4-$10\,000\,min^{-1}$.
Inhibition angle sensitivity	β	22	
**Nucleator parameters**			
Number of Nucleators	N	10000-12000	Explored range: 100-12000 and N=850 for stable simulations
Nucleator binding-branching rate	k_b	$2.8 \times 10^{-3}\,\mu m^{-1}\,min^{-1}$	See section 3.2
Nucleating angle std	σ_{bra}	$0.157\,rad$	
**Centrosome parameters**			
Nucleation rate	k_n	$30\,min^{-1}$	Explored range: 6-3000 min^{-1} and $1200\,min^{-1}$ for stable simulations
Centrosome radius	R_c	$0.1\,\mu m$	
**Domain parameters**			
Box length (X and Y)	L	$313\,\mu m$	$94\,\mu m$ for stable simulations
Box height (Z)	L_z	$50\,\mu m$	
**Integration parameters**			
Timestep	Δt	$0.01\,s$	Chosen for accuracy and efficiency
Snapshot period	t_{snap}	$10\,s$	
Total time	T	45-120 min	

Supplementary Table 7: Parameters of 3D simulations of asters. Physical parameters not measurable through experiments were chosen to give the best fit to experimental data.
