# Supplementary material for: Robust cytoplasmic partitioning by solving a cytoskeletal instability
Source: Nature. 2026 Jan 28;651(8105):501–10. doi: 10.1038/s41586-025-10023-z (PMC12979205; doi:10.1038/s41586-025-10023-z)
Supplement: Supplementary file 2 — Reporting Summary [file 41586_2025_10023_MOESM2_ESM.pdf]

nature portfolio

Corresponding author(s): J. Brugués and M. Rinaldin

Last updated by author(s): 23/10/2025

Reporting Summary

Nature Portfolio wishes to improve the reproducibility of the work that we publish. This form provides structure for consistency and transparency in reporting. For further information on Nature Portfolio policies, see our Editorial Policies and the Editorial Policy Checklist.

Statistics

For all statistical analyses, confirm that the following items are present in the figure legend, table legend, main text, or Methods section.

n/a	Confirmed	
☐	☒	The exact sample size (*n*) for each experimental group/condition, given as a discrete number and unit of measurement
☐	☒	A statement on whether measurements were taken from distinct samples or whether the same sample was measured repeatedly
☐	☒	The statistical test(s) used AND whether they are one- or two-sided<br>*Only common tests should be described solely by name; describe more complex techniques in the Methods section.*
☒	☐	A description of all covariates tested
☒	☐	A description of any assumptions or corrections, such as tests of normality and adjustment for multiple comparisons
☐	☒	A full description of the statistical parameters including central tendency (e.g. means) or other basic estimates (e.g. regression coefficient) AND variation (e.g. standard deviation) or associated estimates of uncertainty (e.g. confidence intervals)
☒	☐	For null hypothesis testing, the test statistic (e.g. *F*, *t*, *r*) with confidence intervals, effect sizes, degrees of freedom and *P* value noted<br>*Give P values as exact values whenever suitable.*
☐	☒	For Bayesian analysis, information on the choice of priors and Markov chain Monte Carlo settings
☒	☐	For hierarchical and complex designs, identification of the appropriate level for tests and full reporting of outcomes
☒	☐	Estimates of effect sizes (e.g. Cohen's *d*, Pearson's *r*), indicating how they were calculated

Our web collection on statistics for biologists contains articles on many of the points above.

Software and code

Policy information about availability of computer code

Data collection	The Method section specifies how the following microscopes were used for acquiring specific data sets. Confocal data were acquired with:<br>(1) a spinning disk confocal microscope (IX83 Olympus microscope with a CSU-W1 Yokogawa disk) connected with two Hamanatsu ORCA-Fusion BT Digital CMOS camera), equipped with photoactivation module, and Olympus cellSens 4.3.1 software.<br>(2) a spinning disk confocal microscope Olympus IXplore SpinSR with cellSens 4.3.1 and Andor Revolution Spinning disk with Andor iQ3.6 software.<br>(3) a laser scanning confocal microscope Leica SP8 microscope with a Leica Application Suite X (LAS X). The FRAP 4 Module was used for FRAP experiments.<br>(4) a spinning confocal microscope Andor IX 81 microscope with a Yokogawa CSUX1 spinning disk and Andor iQ 3.6 software. The FRAP module FRAPPA was used for FRAP experiments.<br>Light sheet data were acquired with a Zeiss LightSheet Zeiss Z.1 and ZEN 2014 SP1 v9.2.10.54 software.
Data analysis	Data sets were analyzed with Fiji ImageJ2 Version 2.14.0/1.54f and custom made scripts in Python (version 3.9) runnig on PyCharm 2021.1.3. In Fiji, the following plugins were used: Substract Background to remove background, StackReg for image registration for invasion time estimation, Trackmate (PMID: 35654950) run on a custom-made Jython script for tracking of EB1 comets and speckles, 3D Viewer for 3D image reconstruction. All maximum intensity projections were performed in Fiji. Linear density profiles were measured in Fiji with PlotProfile function. Mean values of intensities, lengths and areas of objects of interest were measured with SetMeasurement and Measure function in Fiji. Cellpose 2.0 (PMID: 36344832) was used to segment compartments and it was run on a Python script.

For manuscripts utilizing custom algorithms or software that are central to the research but not yet described in published literature, software must be made available to editors and reviewers. We strongly encourage code deposition in a community repository (e.g. GitHub). See the Nature Portfolio guidelines for submitting code & software for further information.

Data

Policy information about availability of data

All manuscripts must include a data availability statement. This statement should provide the following information, where applicable:

- Accession codes, unique identifiers, or web links for publicly available datasets
- A description of any restrictions on data availability
- For clinical datasets or third party data, please ensure that the statement adheres to our policy

There is no restriction on data availability. Source data are provided with the online version of this paper and raw data are deposited on the online repository:

Research involving human participants, their data, or biological material

Policy information about studies with human participants or human data. See also policy information about sex, gender (identity/presentation), and sexual orientation and race, ethnicity and racism.

Reporting on sex and gender	*Use the terms sex (biological attribute) and gender (shaped by social and cultural circumstances) carefully in order to avoid confusing both terms. Indicate if findings apply to only one sex or gender; describe whether sex and gender were considered in study design; whether sex and/or gender was determined based on self-reporting or assigned and methods used. Provide in the source data disaggregated sex and gender data, where this information has been collected, and if consent has been obtained for sharing of individual-level data; provide overall numbers in this Reporting Summary. Please state if this information has not been collected. Report sex- and gender-based analyses where performed, justify reasons for lack of sex- and gender-based analysis.*
Reporting on race, ethnicity, or other socially relevant groupings	*Please specify the socially constructed or socially relevant categorization variable(s) used in your manuscript and explain why they were used. Please note that such variables should not be used as proxies for other socially constructed/relevant variables (for example, race or ethnicity should not be used as a proxy for socioeconomic status). Provide clear definitions of the relevant terms used, how they were provided (by the participants/respondents, the researchers, or third parties), and the method(s) used to classify people into the different categories (e.g. self-report, census or administrative data, social media data, etc.) Please provide details about how you controlled for confounding variables in your analyses.*
Population characteristics	*Describe the covariate-relevant population characteristics of the human research participants (e.g. age, genotypic information, past and current diagnosis and treatment categories). If you filled out the behavioural & social sciences study design questions and have nothing to add here, write "See above."*
Recruitment	*Describe how participants were recruited. Outline any potential self-selection bias or other biases that may be present and how these are likely to impact results.*
Ethics oversight	*Identify the organization(s) that approved the study protocol.*

Note that full information on the approval of the study protocol must also be provided in the manuscript.

Field-specific reporting

Please select the one below that is the best fit for your research. If you are not sure, read the appropriate sections before making your selection.

☒ Life sciences ☐ Behavioural & social sciences ☐ Ecological, evolutionary & environmental sciences

For a reference copy of the document with all sections, see nature.com/documents/nr-reporting-summary-flat.pdf

Life sciences study design

All studies must disclose on these points even when the disclosure is negative.

Sample size	We did no perform statistical analyses to calculate sample size. We chose the sample size based on similar data sets used in the field, reproducibility of the experiments (i.e. experiments was repeated enough time to observe consistent phenotypes), and experimental challenges. We report the sample sizes whenever there are error bars in the legend. We report here below and in the "Statistics and Reproducibility" in the Methods the sample sizes associated to the representative images chosen in the paper.
Data exclusions	Data were excluded in the case embryos or extract underwent early apoptosis.
Replication	Experiments were replicated over the course of about three years with different microscopes and for some conditions by different experimenters (cell cycle arrest in extract by M.R. and other people in Brugués group, cell cycle arrest in Drosophila by M.R and Y. X., cell cycle arrest zebrafish by M. R. and A. K, EB1 comets for zebrafish by M. R. and A. K.). TThe number of biological replicates is indicated as number of independent samples and it refers to independent experiments for the in vitro extract studies and embryo number for in vivo studies. We report these numbers in the legend for plots with error bars and histograms. For the microtubule density profiles (Figure1l, 3c,e,g, 4e,k, and Extended Data Fig. 8 b,e,f,h), number of independent samples are reported on Supplementary Table 1. For measurements of microtubule dynamics (Figure 3a, 4c-d, and Extended Data Fig. 9c), they are reported on Supplementary Tables 1-3. Technical replicates are reported in the adjacent columns. In the Methods section and here, we provide the number of replicates for images representative of phenotypes in the

	Main Text and Extended Data Figures: Figure1: (b)Imaging of the microtubules and actin first cell cycle in zb embryo was repeated n>20 times with confocal spinning disk and n=1 with light sheet for visualization purposes. (c) n=4. (d) n=8. (e,f) n > 20. (g) n=4. (h) n=8. (i,j,k) n>20. Figure 2: (a,b) Experiments with invasion events were repeated n>20. Figure 3: (d,f) n>5. (h) n=8. (i) n=4. Figure 4: (a,b,f,l) n>20. (j) n=1 specifically with confinement in droplets, but n>20 to test morpholinos in extract. Figure 5: The specific videos were acquired as n=1 as proof of concept, however these dynamics were observed n>20. Extended Data Fig. 1: (b) n=8. (c) n=4. (e) n=1. (f) n=12. (h,i) n>20. (k) n=8. Extended Data Fig. 2: (g) n>20. (h) n=3. Extended Data Fig. 4: (a,e) n>5. (d) n=3. Extended Data Fig. 5: n>5.Extended Data Fig. 6: Experiments with invasion events were repeated n>20. This is a representative analysis of the event. (e) n>5. (f) n=3. Extended Data Fig. 7: (a) Experiments with invasion events were repeated n>20. This is a representative analysis of the event to show the method to find the invasion time. Extended Data Fig. 8: : (c,d) n=6. (g) n=2. (i,j) n>5. Extended Data Fig. 10: (a) n=11. (b) n= 12. (c) n=9. (g) n=8. (h) n=14. (i) n>5. Plots showing single lines without errors are relative to specific images or video (Figure 5e in the Main Text and Extended Data Figures 1b,d,g,j, 2c, 4d,g, 6b-e, 7b-d,10 d-e). These plots are based on the quantification of a single example of a phenotype that was replicated with the n value related for the figures and reported above.
Randomization	In the analysis of the role of cell cycle times (Figure 2g-h) and invasion times (Figure 2d), experiments were analyzed in a random order as there was no prior knowledge on the possible outcome. For the other experiments, randomization was not performed and each condition was analyzed separately.
Blinding	We did not perform blinding.

Reporting for specific materials, systems and methods

We require information from authors about some types of materials, experimental systems and methods used in many studies. Here, indicate whether each material, system or method listed is relevant to your study. If you are not sure if a list item applies to your research, read the appropriate section before selecting a response.

Materials & experimental systems

n/a	Involved in the study
☐	☒ Antibodies
☐	☒ Eukaryotic cell lines
☒	☐ Palaeontology and archaeology
☐	☒ Animals and other organisms
☒	☐ Clinical data
☒	☐ Dual use research of concern
☒	☐ Plants

Methods

n/a	Involved in the study
☒	☐ ChIP-seq
☒	☐ Flow cytometry
☒	☐ MRI-based neuroimaging

Antibodies

Antibodies used	Anti-INCENP (abcamp ab12183) and Anti Aurora kA (gift of Keisuke Ishihara (PMID: 27892852)
Validation	Anti-INCENP was labelled with Alexa 14 Fluor 488 NHS Ester (Thermofisher, A20000), tested in extract and and zebrafish embryo for localization in the Chromosome Passenger Complex. Anti Aurora kA was used to coat beads and tested for microtubule nucleation.

Eukaryotic cell lines

Policy information about cell lines and Sex and Gender in Research

Cell line source(s)	Wild type Hela cells were used for centrosome purification and were obtained from the Hyman Lab
Authentication	The cells were authenticated in March 2023 with PCR by Eurofins
Mycoplasma contamination	The cells tested negative or Mycoplasma on 16.12.20 and 30.4.21
Commonly misidentified lines (See ICLAC register)	This study did not involve ICLAC lines.

Animals and other research organisms

Policy information about studies involving animals; ARRIVE guidelines recommended for reporting animal research, and Sex and Gender in Research

Laboratory animals	Xenopus Laevis, Drosophila melanogaster, and Danio rerio.
Wild animals	This study did not involve wild animals.
Reporting on sex	Sex identification cannot be performed for the early embryos in this study (max a few hours aster fertilization).

Field-collected samples	We did not collect samples in the field.
Ethics oversight	Experiments with Xenopus laevis were approved and licensed by the local animal ethics committee (Landesdirektion Sachsen, Germany; license no. DD24-5131/367/9, 25-5131/521/12, and 25-5131/564/25) and carried out following the European Communities Council Directive 2010/63/EU on the protection of animals used for scientific purposes, as well as the German Animal Welfare Act. Experiments with Danio rerio were approved and licensed by the local animal ethics committee (Landesdirektion Sachsen, Germany; license no. DD24.1-5131/ 394/ 33) and carried out following he European Communities Council Directive 2010/63/EU on the protection of animals used for scientific purposes, as well as the German Animal Welfare Act. No ethics oversight was necessary for experiments with Drosophila melanogaster.

Note that full information on the approval of the study protocol must also be provided in the manuscript.

Plants

Seed stocks	*Report on the source of all seed stocks or other plant material used. If applicable, state the seed stock centre and catalogue number. If plant specimens were collected from the field, describe the collection location, date and sampling procedures.*
Novel plant genotypes	*Describe the methods by which all novel plant genotypes were produced. This includes those generated by transgenic approaches, gene editing, chemical/radiation-based mutagenesis and hybridization. For transgenic lines, describe the transformation method, the number of independent lines analyzed and the generation upon which experiments were performed. For gene-edited lines, describe the editor used, the endogenous sequence targeted for editing, the targeting guide RNA sequence (if applicable) and how the editor was applied.*
Authentication	*Describe any authentication procedures for each seed stock used or novel genotype generated. Describe any experiments used to assess the effect of a mutation and, where applicable, how potential secondary effects (e.g. second site T-DNA insertions, mosiacism, off-target gene editing) were examined.*
