# Supplementary material for: Author Correction: Robust cytoplasmic partitioning by solving a cytoskeletal instability
Source: Nature. 2026 Apr 22;653(8113):E1. doi: 10.1038/s41586-026-10390-1 (PMC13149297; doi:10.1038/s41586-026-10390-1)

nature portfolio

https://doi.org/10.1038/s41586-026-10390-1

Supplementary information

Author Correction: Robust cytoplasmic partitioning by solving a cytoskeletal instability

In the format provided by the authors and unedited

Supplementary Information to Author Correction: Robust cytoplasmic partitioning by solving a cytoskeletal instability

Melissa Rinaldin, Alison Kickuth, Adam Lamson, Benjamin Dalton, Yitong Xu, Pavel Mejstřík, Stefano Di Talia & Jan Brugués

Correction to: *Nature* https://doi.org/10.1038/s41586-025-10023-z. Published online 28 January 2026.

Figure 1l, original

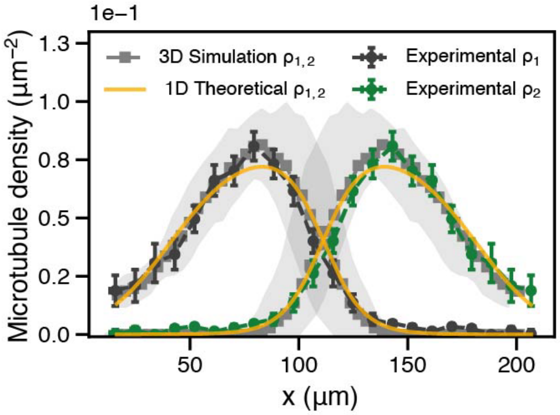

Figure 1l, corrected

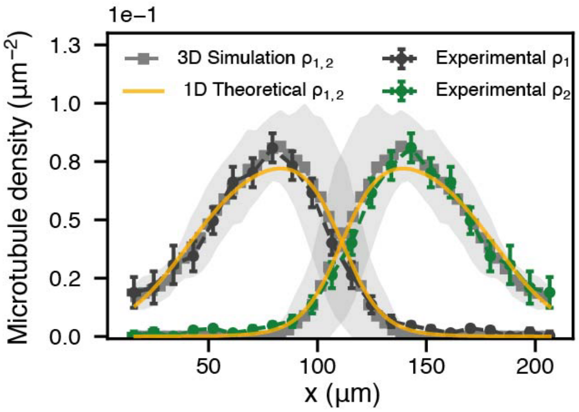

Extended Data Figure 8b, original

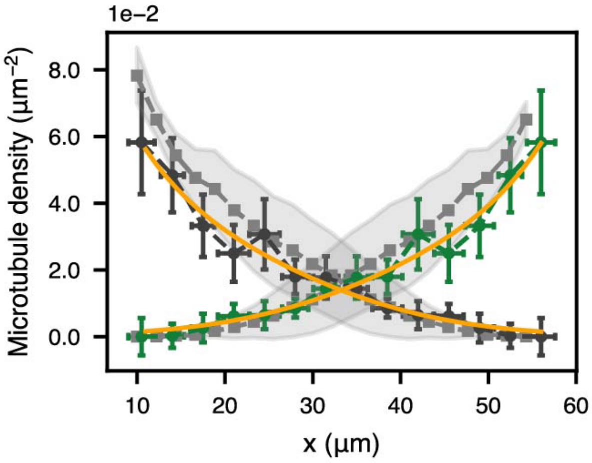

Extended Data Figure 8b, corrected

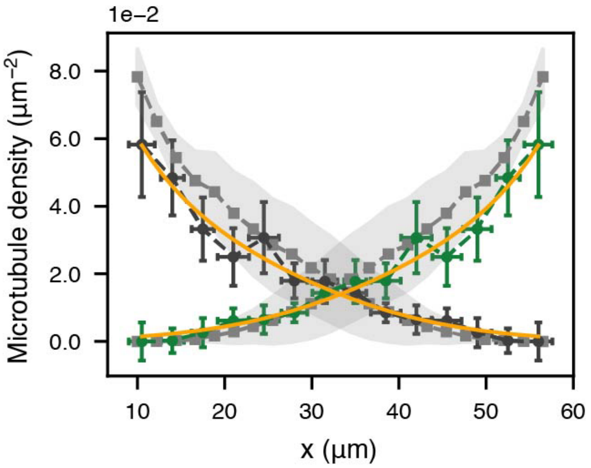

Supplement: Supplementary file 1 — Original and revised Fig. 1l and Extended Data Fig. 8b [file 41586_2026_10390_MOESM1_ESM.pdf]
